# Correction: Cao et al. Tubeimoside-1 Inhibits Glioblastoma Growth, Migration, and Invasion via Inducing Ubiquitylation of MET. *Cells* 2019, *8*, 774

**DOI:** 10.3390/cells13181577

**Published:** 2024-09-19

**Authors:** Jiangjun Cao, Erhu Zhao, Qingzong Zhu, Juanli Ji, Zekun Wei, Bo Xu, Hongjuan Cui

**Affiliations:** 1 Chongqing Engineering Research Center of Antitumor Natural Drugs, Chongqing Three Gorges Medical College, Chongqing 404120, China; jiangjuncao@163.com; 2 State Key Laboratory of Silkworm Genome Biology, Southwest University, Chongqing 400715, China; erhuzhao@126.com (E.Z.); zqz26113929@outlook.com (Q.Z.); juanli0410@126.com (J.J.); weizekun00@163.com (Z.W.); 3 Institute of Medicine of Southwest University, Southwest University, Chongqing 400715, China

## Author’s Information

In the Correspondence information of the original publication [1], there was an error related to the contact telephone numbers. The telephone numbers were deleted.

## Error in Figures

In the original publication, there were mistakes in Figure 3 and Figure 4 as published. In Figure 3c, ‘Invision’ was spelled wrong, and it was replaced by ‘Invasion’. Furthermore, ‘* *p* < 0.05’ is not mentioned and it was deleted from the caption. Also, the LN229/Invasion group in the same subfigure presented an overlap in both DMSO/Invasion and 5 µM/Invasion. In Figure 4a, the protein band of p-AKT was incorrectly used in the U87 cell line.

**Figure 3:**
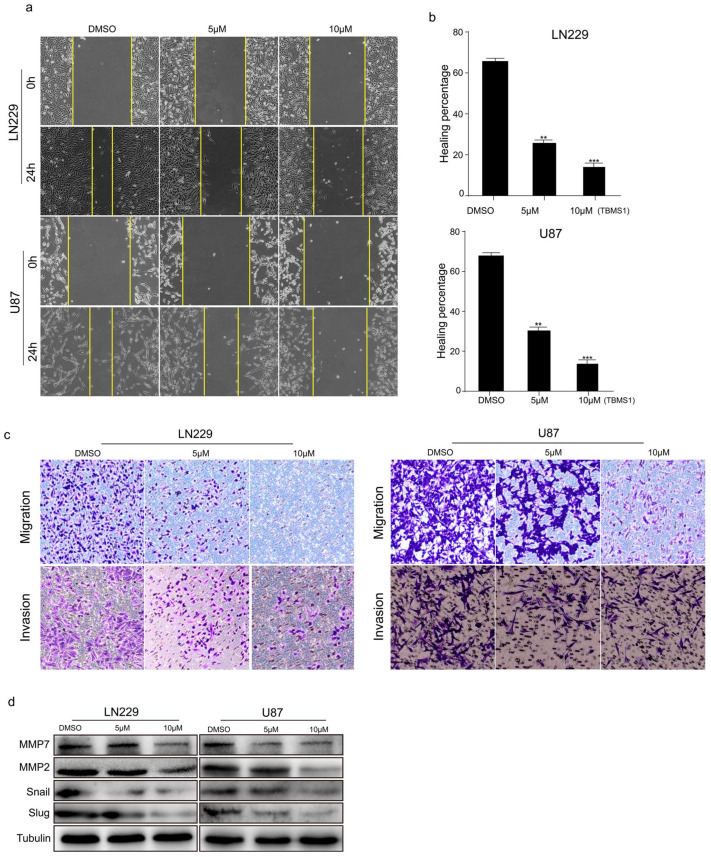
TBMS1 blocks glioblastoma cells migration and invasion. (**a**) The migration ability of glioblastoma cells treated with different concentrations of TBMS1 was measured by wound-healing assay. DMSO was added as a control. (**b**) According to (**a**) plot, the percentage of cell healing was counted under different TBMS1 concentrations. (**c**) The invasion ability of glioblastoma cells treated with different concentrations of TBMS1 was measured by Transwell assay. DMSO was added as a control. (**d**) The expression levels of MMP7, MMP2, Snail, Slug, and Tubulin proteins in LN229 and U87 cells were measured by western blot treated with different concentrations of TBMS1. DMSO was added as a control. ** *p* < 0.01, *** *p* < 0.001, *p*-values < 0.05 were considered as statistically significant.

**Figure 4:**
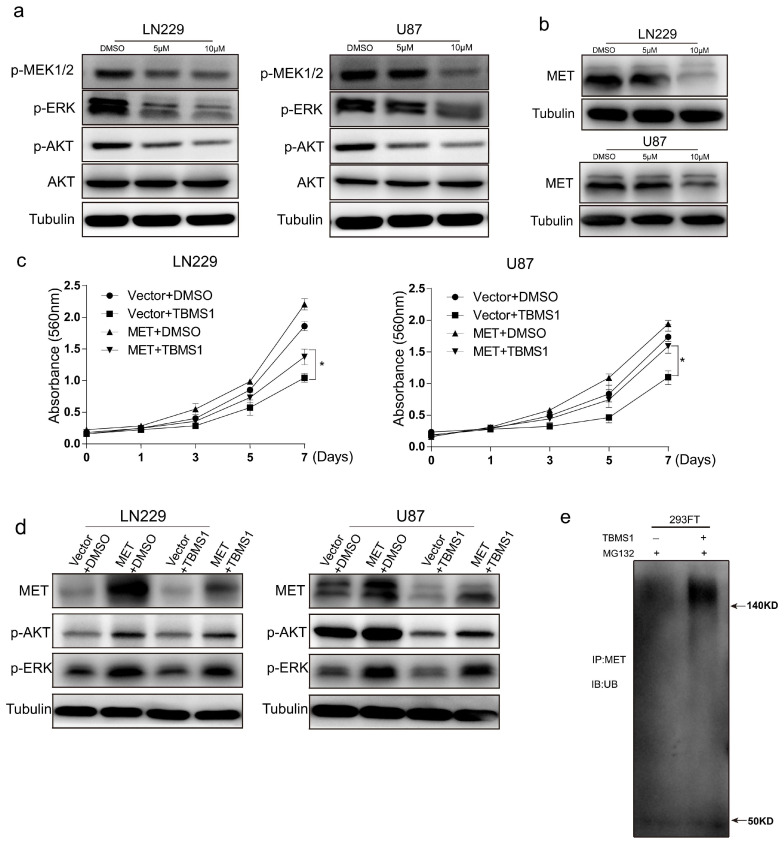
TBMS1 inhibits glioblastoma cells activation of AKT, ERK pathway by enhancing MET ubiquitination degradation. (**a**) The expression levels of p-MEK, p-ERK, p-AKT, AKT, and Tubulin proteins in LN229 and U87 cells were measured by western blot treated with different concentrations of TBMS1. DMSO was added as a control. (**b**) The expression levels of MET and Tubulin proteins in LN229 and U87 cells were measured by western blot treated with different concentrations of TBMS1. DMSO was added as a control. (**c**) Viability of LN229 and U87 cells overexpressing MET and vector under treatment with TBMS1 (5 μM), respectively. DMSO was added as a control. (**d**) The expression levels of MET, p-AKT, p-ERK and Tubulin proteins in LN229 and U87 cells overexpressed MET and vector respectively were measured by western blot. Cells were supplemented with TBMS1 (5 μM) and DMSO before the experiment. (**e**) The level of ubiquitination of the MET protein in 293FT cells after the addition of TBMS1 (5 μM) was examined using an IP assay. DMSO was added as a control. * *p* < 0.05, *p*-values < 0.05 were considered as statistically significant.

The correct figures appear below. The authors state that the scientific conclusions are unaffected. This correction was approved by the Academic Editor. The original publication has also been updated.
